# Immunological and virological characterization of HIV-1 viremia controllers in the North Region of Brazil

**DOI:** 10.1186/s12879-017-2491-9

**Published:** 2017-06-01

**Authors:** Samara Tatielle M. Gomes, Érica R. Gomes, Mike B. dos Santos, Sandra S. Lima, Maria Alice F. Queiroz, Luiz Fernando A. Machado, Izaura M. V. Cayres-Vallinoto, Antonio Carlos R. Vallinoto, Marluísa de O. Guimarães Ishak, Ricardo Ishak

**Affiliations:** Federal University of Para, Institute of Biological Sciences, Virus Laboratory, Campus Belem, Belem, Para 66000–000 Brazil

**Keywords:** HIV-1, Viral controllers, Elite controllers, Cytokines, CCR5, SDF1

## Abstract

**Background:**

A rare phenotype of clinical non-progressors to AIDS is not well understood and the new protocol for universal treatment, may block the understanding of viral control thus it is crucial to define this controversial group.

**Methods:**

A cohort of 30 persons followed a criteria for viremia control groups 1 (VC1; *n* = 2) and 2 (VC2; *n* = 7) and non-viral controllers (NC; *n* = 21) including number of years of diagnosis, LTCD4^+^, LTCD8^+^ counts, plasma viral load and the absence of ART; 241 uninfected control persons were matched to age and sex. Infected persons were regularly examined and submitted to two or three annual laboratory measurements. Polymorphisms and allele frequencies of CCR5Δ32 and SDF1–3’A were detected in the genomic DNA. Plasma levels of cytokines (IL-2, IL-4, IL-5, IL-9, IL-10, IL-13, IL-17 and IFN-y) were measured.

**Results:**

The group investigated is originated from a miscigenetic population and demographic and social characteristics were not significantly relevant. LTCD4^+^ median values were higher among VC than NC, but significantly lower than uninfected controls. Evolution of LTCD4^+^ and LTCD8^+^ counts, showed a slight increase of LTCD4^+^ among VC, but a significant decrease in the NC. The percentage of annual change in LTCD4^+^ was also significantly different between the groups. LTCD4^+^/LTCD8^+^ ratio was inverted but not significant among the VC, thus the ratio may be a useful biomarker for the VC. A clear signature indicated a change from Th1 to Th2 cytokine profiles from VC to NC, respectively.

**Conclusions:**

The knowledge of viral controllers characteristics in different population groups is important to define a strict universal definition for the sake of learning about the pathogenesis of HIV-1. Data on LTCD4^+^ seems to be stable and repetitive from published data, but the LTCD8^+^ response and the significance of LTCD4^+^/LTCD8^+^ ratio values are in need to further exploration as biomarkers. The change from Th1 to Th2 cytokine profile may help to design and adjust specific treatment protocols for the group.

## Background

The usual period of slow, but continuous viral replication of HIV-1, T CD4^+^ lymphocyte (LTCD4^+^) decline and the appearance of AIDS followed by death, was the natural history accompanying HIV-1 infecting humans [1, 2]. The use of different protocols which should be strictly followed to treat the viral infection (antiretroviral therapy, ART), made it possible to interfere in the process, although sometimes with several nasty side effects of the therapeutical drugs used [3, 4]. The suggestions of clinical and laboratory evidences that a small number of HIV-1 infected persons could be able to naturally control the virus without the use of anti-retroviral therapy, opened a window of hope to millions of persons carrying the enormous burden of a deadly infection [5–7].

A new phenotype of HIV-1 carriers, the long term non-progressor, elite controller or viral controller, among others, define a group of HIV-1 infected persons who could naturally maintain viral replication under control and the absence of precipitating events which could lead to AIDS [8–11]. The definition of viral controller and, particularly, elite controller, is not equally defined and there is no universal consensus as can be seen in the several different proposals [10, 12–14]. The suggestion of the possibility of HIV-1 eradication or the maintenance of a host who would be non infectious, is a bold proposal considering the retrospective and accumulated knowledge in virus strategies of replication, adaptation to the host and perpetuation together with the infected host [15–17].

There was a recent change in the time to start treatment of infected persons as soon as the infection is diagnosed which was also adopted in Brazil [18]. The recent evidences of three large and well conducted studies (HPTN 052, Temprano and START), were sufficiently strong to support the change, once more, in the usual protocol which define the starting point to treatment [19–21]. The “test and treat” approach may lead to a difficulty to recognize those patients who are able to maintain the virus under natural control and make it impossible to learn how the process can be attained. Here we present the characterization of the human model currently described in the city of Belem, Para (Northern Brazil), in order to provide additional criteria to turn the definition of this controversial point of discussion even more strict and to start collaborative studies in the country, aiming to better understand this phenotype of viral controllers. It is important to tighten the characterization of the subjects in order to make sure we can really believe in the control and a possible future cure of HIV-1 infection.

## Methods

### Criteria to define the groups involved

The classification criteria for the viremia control 1 and 2 (VC1 and VC2), and the non-viral controller (NC) groups, used information related to the number of years of known diagnosis, LTCD4^+^ and LTCD8^+^ counts and plasma viral load quantification are described in Table 1. It is important to emphasize that all persons were aware of their status of HIV-1 carriers for 6 years or more and were clinically and laboratory followed with their measurements of LTCD4^+^, LTCD8^+^ and plasma viral load at least twice a year. All of the three groups were treatment naïve and the start of ART at any time during the period of observation was the only exclusion criteria. The initial LTCD4^+^, LTCD8^+^ counts and their rate were considered to be the baseline values; the latest was the last value recorded during the period of observation (from January 2007 to December 2013).

**Table 1:** Definition criteria of viral controller and non-viral controller groups investigated, according to the time of infection without ART, immunological and virological measurements

Groups according to disease progression	Time of known infection	Time of data collection	Use of antiretroviral therapy	Measurements of T CD4^+^ lymphocytes	Measurements of HIV-1 viral load	Important remarks
VC1: Viremia Controller 1 (Elite Controller)	>6 years	Since 2007 (minimum of 3 years of data)	No ART	>500 cells/mm^3^	<50 copies/mL (not detectable)	No episodes of increase in viral load; counts of LTCD4^+^ greater than 500 cells/mm^3^; maintains stability for more than 6 years; no ART intervention
VC2: Viremia Controller 2	>6 years	Since 2007 (minimum of 3 years of data)	No ART	>500 cells/mm^3^	≤ Log_10_ 4 (up to 10,000 copies/mL)	Episodes of increase in viral load and decrease in counts of LTCD4^+^, independent of the number of occurrences, but with natural remission, representing no more than 50% of measurements; maintain control for more than 6 years without ART intervention
NC: Non-Viremia Controller	>6 years	Since 2007 (minimum of 3 years of data)	No ART	<500 cells/mm^3^	> Log_10_ 4 (greater than 10,000 copies/mL)	

A cohort of 30 persons was distributed in three groups, VC1 (*n* = 2), VC2 (*n* = 7) and NC (*n* = 21). To each person investigated, there was a group of seven to nine uninfected match controls, regarding sex and age (range of 5 years up or down) of regular blood donors from the Fundacao HEMOPA. To the VC1 there were 19 persons (nine women and ten men), to VC2, 55 (20 women and 35 men) and NC, 167 (91 women and 76 men) controls. The composition of the cohort according to sex and age is described in Table 2.

**Table 2:** Population composition of the three groups (viral and non-viral controllers) according to sex and age

Groups	Males *n* (%)	Females *n* (%)	Age range Males	Age range Females
VC1	1 (50)	1 (50)	52	45
VC2	3 (43)	4 (57)	34–47	35–53
NC	11 (52)	10 (48)	28–68	24–68

*VC1* Viremia Controller 1 (Elite Controller), *VC2* Viremia Controller 2, *NC* Non-Viremia Controller

### Routine examination

The groups were routinely examined by a clinician according to the Brazilian national follow up program of HIV-1 infected persons in which they have included two to three annual measurements of HIV-1 plasma viral load and LTCD4^+^/LTCD8^+^ counts. Viral load was initially measured using branched DNA methodology (bDNA - Bayer Corporation, Massachusetts, USA) and presently uses a Real Time PCR methodology (Abbott Molecular, USA); in both the limit of detection was 50 copies/mL. Lymphocyte measurements were performed using flow citometry (Becton-Dickson, USA). Both methods followed the manufacturer’s recommendation.

### Polymorphisms of CCR5Δ32 and SDF1–3’A

Genomic DNA was purified from peripheral blood cells using a commercial kit (QIAamp DNA Mini Kit, QIAGEN, Germany). The genotypes of CCR5Δ32 and SDF1–3’A were determined as previously described [22, 23]. Forward and reverse primers were: F-5’GTCTTCATTACACCTGCAGCTCT-3′ and R-5′-CACAGCCCTGTGCCTCTT-3′ for CCR5Δ32, F-5’CAGTCAACCTGGGCAAAGCC-3′ and R-3′-AGCTTTGGTCCTGAGAGTCC-5 for SDF1–3’A.

The amplification of the wild type allele of CCR5 yields a product of 189 bp, while the mutant allele (CCR5∆32) yields a shorter one (157 bp). Amplification products of SDF1–3′ (302 bp) were digested with restriction endonuclease *MspI* 5 U (New England Biolabs), for 3 hours at 37 °C. In the presence of allele 3’A the product is not cleaved resulting in a fragment of 302 bp, but in the absence of the mutation, the fragment is cleaved in two pieces (202 and 100 bp). The amplified and digested products were seen following electrophoresis (100 V/45 min) in a 3% agarose gel with Sybr Safe, diluted in TAE 1×, using a transilluminator with an ultraviolet light.

### Cytokine measurements

Plasma levels of eight cytokines (IL-2, IL-4, IL-5, IL-9, IL-10, IL-13, IL-17 and IFN- γ) were measured using Luminex® xMAP® in a multiplex assay (Th1/Th2/Th17 Human Magnetic 8-Plex Panel for Luminex™ Platform 200, Invitrogen™, USA) according to the manufacture’s recommendation and read on a Luminex plataform.

### Statistical analysis

Statistical analysis were performed using two softwares, GraphPad Prism, version 5.0 and BioEstat, 5.3 [24]. The association of the groups and sexual behavior was performed using the G test. Construction of the Figures used median values and interquartile range. All of the available counts for each person during the period of study were used for the comparisons. The Mann–Whitney test was used to compare counts of LTCD4^+^, LTCD8^+^ and their ratios among the groups involved. Significance between first and last counts of LTCD4^+^, LTCD8^+^ and their ratio was performed using the Wilcoxon test. The median yearly change in absolute counts of LTCD4^+^, LTCD8^+^ and their ratio was calculated by the compound annual growth rate (CAGR) as previously described [17]. The differences of CAGR between the groups were measured by the Mann–Whitney test. Chi-square and residue analysis were used to evaluate the association of plasma viral load of HIV-1 between VC and NC groups. The polymorphisms and allele frequencies were compared using the G test. The differences of cytokine levels were performed using the Mann–Whitney test. Significance was assumed at the level of 0.05 for all the statistical methods used.

## Results

The age of the persons investigated in the present study, ranged from 24 to 68 years. The group included equal numbers of persons from both sexes and the majority (70%; 21/30) referred to be single (Table 3). Most of the persons (70%) informed to be heterosexual who make use of non-injecting drugs (53.3%; 16/30). Although they did not mention sexual relations with persons who have multiple sexual partners (93.3%; 28/30), 46.7% (14/30) referred to have had sexual relations with HIV-1 infected persons, 56.7% (16/30) do not use condoms and 51.8% (14/27) perform anal sex. None of the involved persons was infected either with HBV or HCV. The individuals in groups VC1 and VC2, referred no promiscuity and no anal sexual relations. None of the above demographic or social characteristics showed a significant statistical level of importance.

**Table 3:** Demographic and social characteristics of the HIV-1 infected groups (viral controller and non-viral controller) investigated

Variables	VC1 *n* (%)	VC2 *n* (%)	NC *n* (%)	*p*
Age (in years)				
20–40	0	4 (57)	10 (48)	
41–61	2 (100)	3 (43)	9 (43)	
62–82	0	0	2 (9)	0.3478
Sex				
Male	1 (50)	3 (43)	11 (52)	
Female	1 (50)	4 (57)	10 (48)	0.9089
Marital Status				
Single	1 (50)	4 (57)	16 (76)	
Married	1 (50)	3 (43)	5 (24)	0.2460
Sexual behavior				
Heterosexual	2 (100)	5 (72)	14 (67)	
Homosexual	0	2 (28)	4 (19)	
Bisexual	0	0	3 (14)	0.4872
Risk Behavior				
Use of non injecting drugs	1 (50)	4 (57)	11 (52)	
Use of injecting drugs	0	0	1 (5)	
No use of drugs	1 (50)	3 (43)	9 (43)	0.9436
Sexual relation with promiscuous partner	0	0	2 (9)	
No relation with promiscuous partner	2 (100)	7 (100)	19 (91)	0.8259
Sexual relation with HIV^+^/AIDS partner	2 (100)	3 (43)	9 (43)	
No sexual relation with HIV^+^/AIDS partner	0	4 (57)	12 (57)	0.7073
Use of condom	1 (50)	2 (29)	10 (48)	
No use of condom^a^	1 (50)	5 (72)	11 (52)	0.9305
Anal sexual relation	0	3 (43)	11 (61)	
No anal sexual relation^b^	2 (100)	4 (57)	7 (39)	0.7347

*VC1* viremia controller 1 (Elite Controller), *VC2* viremia controller 2, *NC* non-viremia controller

^a^Includes the answers “No” and “Sometimes”

^b^Excludes those who refused to answer

**Fig. 1:**
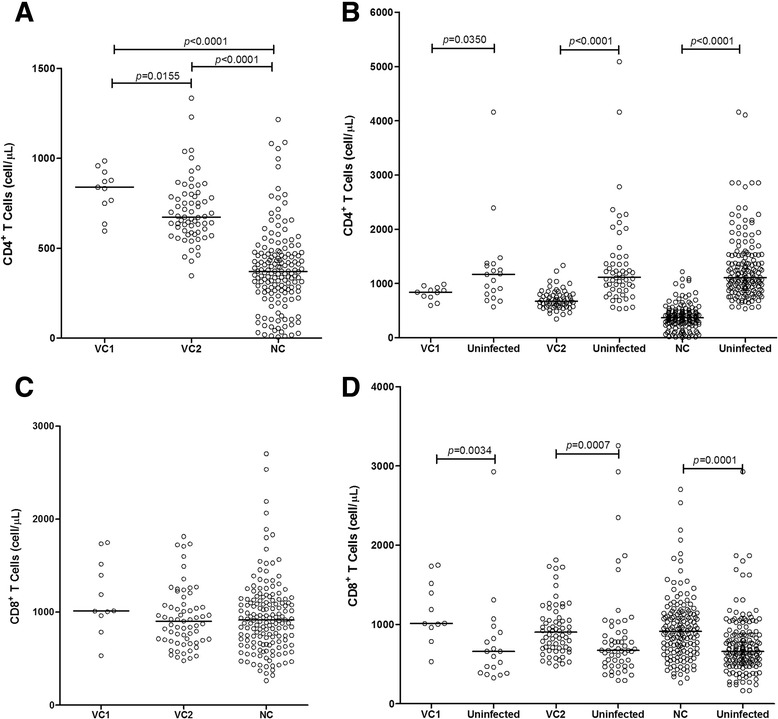
LTCD4^+^ and LTCD8^+^ cell counts among the groups investigated. LTCD4^+^ (**a** and **b**) and LTCD8^+^ (**c** and **d**) cell counts among viral controller (VC1, VC2) and non-viral controller (NC) groups and uninfected persons

**Fig. 2:**
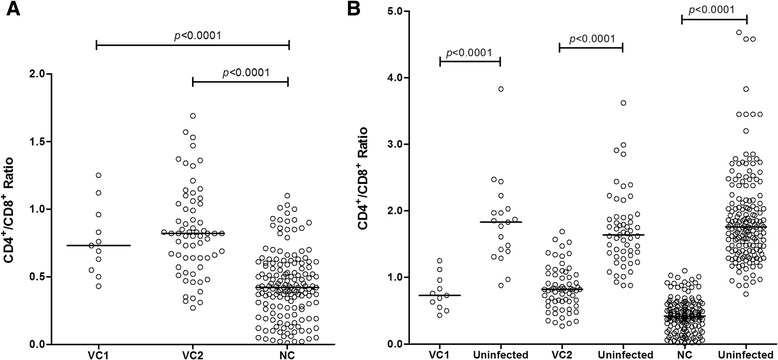
LTCD4^+^/LTCD8^+^ ratio among the groups investigated. LTCD4^+^/LTCD8^+^ ratio among viral controller (VC1, VC2) and non-viral controller (NC) groups (**a**) and uninfected persons (**b**)

Viral controllers (VC1 and VC2) presented a significantly higher count of median values of LTCD4^+^ (839 and 673, respectively) in comparison to the NC (370; *p* < 0.0001), although they were still significantly lower (*p* = 0.0350 and *p* < 0.0001) when compared to their individual uninfected controls (1166, 1114 and 1110, respectively; Fig. 1a and b). LTCD8^+^ values were slightly higher among VC1 (1014), but not significantly different from the other groups (903 and 915.5); the three groups were significantly higher (*p* = 0.0034, *p* = 0.0007 and *p* = 0.0001) than their uninfected controls (660, 674, and 660, respectively; Fig. 1c and d). LTCD4^+^/LTCD8^+^ ratio was significantly higher (*p* < 0.0001) in the VC1 (0.73) and VC2 (0.82) groups as compared to the NC (0.42), but all three were significantly lower (*p* < 0.0001) than the uninfected controls (1.83, 1.64 and 1.76; Fig. 2a and b).

**Fig. 3:**
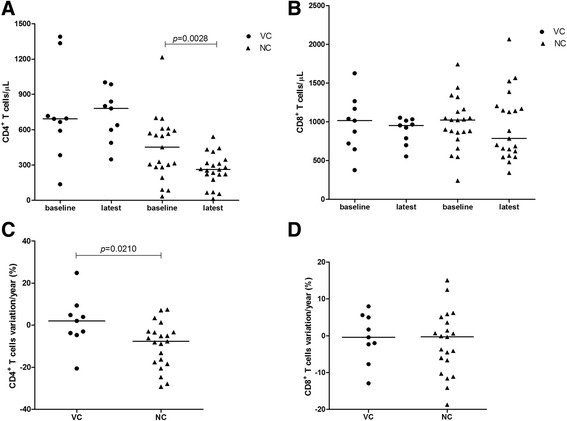
Evolution of LTCD4^+^ and LTCD8^+^ cell counts among viral controller and non-viral controller groups. Evolution of LTCD4^+^ and LTCD8^+^ cell counts among viral controller (VC1 and VC2) and non-viral controller (NC) groups; (**a** and **b**) absolute values; (**c** and **d**) annual changes (in %) in the absolute counts, measured by the CAGR

**Fig. 4:**
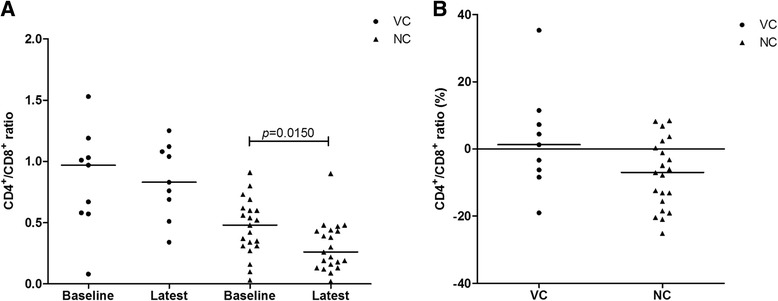
Evolution of LTCD4^+^/LTCD8^+^ ratio among viral controller and non-viral controller groups. Evolution of LTCD4^+^/LTCD8^+^ ratio among viral controller (VC1 and VC2) and non-viral controller (NC) groups; (**a**) absolute values; (**b**) annual changes (in %) in the absolute counts, measured by the CAGR

The evolution of LTCD4^+^ and LTCD8^+^ counts since their first laboratory examination until the end of the period of examination, showed a slight increase (without statistical significance) of LTCD4^+^ among the VC, but a significant (*p* = 0.0028) decrease of the NC (Fig. 3a). The percentage of annual change in LTCD4^+^ was also significantly different between the two groups (Fig. 3c). No difference was observed measuring LTCD8^+^ counts or estimating their annual change (Fig. 3b and d). LTCD4^+^/LTCD8^+^ ratio decreased with time. The inversion of the ratio was not significant among the VC, but significant among the NC, despite there was no significance of their annual change in either group (Fig. 4a and b).

HIV-1 plasma viral load of VC1 was consistently below the limit of the tests used for detection of viral nucleic acid along the observation period. Table 4 shows the frequency distribution of CV (CV1 and CV2) and NC according to four categories, ranging from undetectable to values greater than log_10_ 3 (>10,000 copies/mL). The frequency of undetectable values and of those with lower counts (up to log_10_ 2) were significantly different between the two groups (*p* values >1.96, indicating that there is a lower number of persons than expected); the frequency of higher viral loads were significantly different (*p* values <1.96, indicating that there is a higher number of persons than expected) among the NC group, as measured by the residue analysis.

**Table 4:** Frequency of plasma viral load according to the HIV-1 infected groups (viral controller and non-viral controller) investigated

Viral load	VC (1 + 2)	%	NC	%	χ^2^ (*p*)
<50	13 (+)	22	8 (−)	5	57.01 (<0.0001)
51–1000	19 (+)	32	14 (−)	10	
1001–10,000	28 (+)	46	45(−)	32	
>10,000	0 (−)	0.0	75 (+)	53	
Total	60	100	142	100	

Residue analysis (for significance level, values should be >1.96 or <−1.96; alpha level 0.05); (+) significant positive association; (−) significant negative association; VC (1 + 2): viremia controller 1 and viremia controller 2

*NC* non-viremia controller

**Fig. 5:**
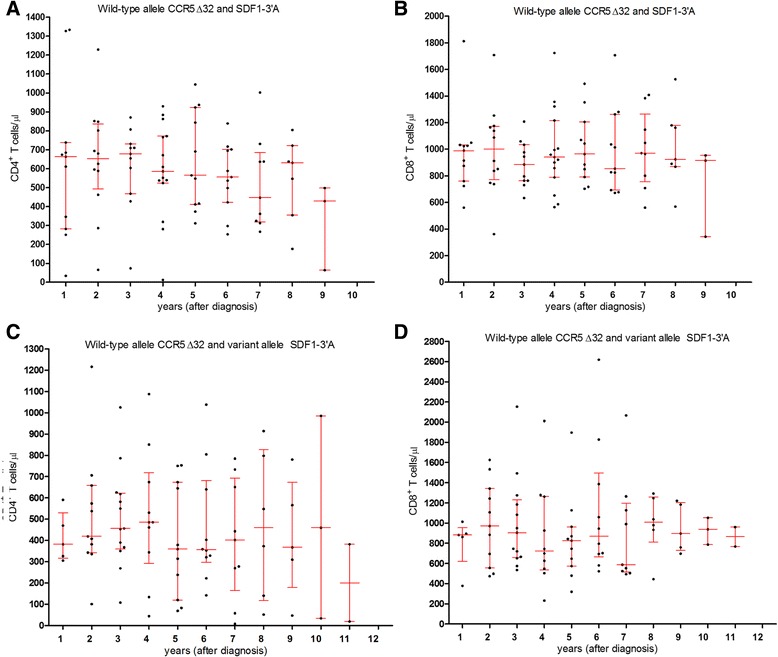
LTCD4^+^ and LTCD8^+^ counts, according to polymorphisms of CCR5Δ32 and SDF1–3’A. LTCD4^+^ (**a** and **c**) and LTCD8^+^ (**b** and **d**) counts, among wild-type allele individuals for CCR5Δ32 and SDF1–3’A and individuals with allele variant allele of SDF1–3’A

None of the individuals presented the CCR5∆32^−^ allele in homozygosis, but it was present in heterozygosis in one individual of the NC group (Table 5). SDF1–3’A polymorphisms were present in hetero and homozygosis among the three groups. No statistical significance was observed either when comparing genotypes or allele frequencies of the three groups. LTCD4^+^ and LTCD8^+^ counts were compared according to the CCR5∆32^−^ and SDF1–3’A polymorphisms. Wild type alleles (Fig. 5a and c) showed median values of LTCD4^+^ > 500 lymphocytes/μL up to 6 years after the initial diagnosis of infection, but in the presence of the allele 3’A the values were consistently lower, during the period of observation; in 36% of the measurements, they were <350 lymphocytes/μL. LTCD8^+^ median counts (Fig. 5b and d) were consistently <1000 lymphocytes/μL along the years and no significant difference was observed, despite the presence of either allele; it is relevant to mention that counts <600 lymphocytes/μL, were more commonly seen (24%) among those with the 3’A allele than among those with the wild type (8%).

**Table 5:** Frequency distribution of genotype and allele frequencies of CCR5∆32 and SDF1–3’A polymorphisms

Genetic profile	VC1 *n* (%)	VC2 *n* (%)	NC *n* (%)	*p*
CCR5 Δ32 variant				
CCR5 Δ32 +/+ (Wild type)	2 (100)	7 (100)	20 (95)	
CCR5 Δ32 −/+ (Heterozygosis)	0	0	1 (5)	
CCR5 Δ32 −/− (Mutant)	0	0	0	0.9478
*CCR5 Δ32 +	4 (100)	14 (100)	40 (95)	
*CCR5 Δ32 -	0	0	2 (5)	0.8344
SDF-1-3’A variant				
G/G (Wild type)	1 (50)	6 (86)	9 (43)	
G/A (Heterozygosis)	0	1 (14)	11 (52)	
A/A (Mutant)	1 (50)	0	1 (5)	0.1817
*G	2 (50)	12 (86)	29 (69)	
*A	2 (50)	2 (14)	13 (31)	0.6396

^*^refers to the alleles

*VC1* viremia controller 1, *VC2* viremia controller 2, *NC* non-viremia controller

**Fig. 6:**
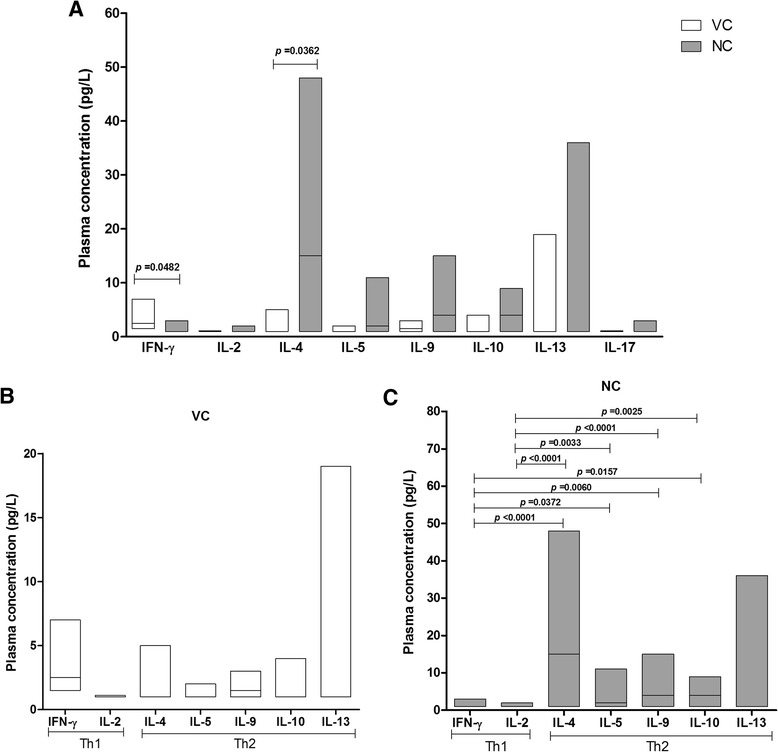
Plasma concentration and comparison of Th1 and Th2 cytokines in the groups investigated. Plasma concentration of cytokines among the VC and NC groups (**a**) and the comparison of Th1 and Th2 cytokines in the two groups (**b** and **c**)

Plasma levels of cytokines IFN-γ, IL-2, IL-4, IL-5, IL-9, IL-10, IL-13 and IL-17, showed that IFN-γ was significantly higher (*p* = 0.0482) in the VC group (Fig. 6a). All the others were higher in the NC group, although only IL-4 was significantly different (*p* = 0.0362). The comparison of Th1 (IFN-γ e IL-2) and Th2 (IL-4, IL-5, IL-10, IL-13) cytokines between the two groups (Fig. 6b and c), showed that IL-2 was present in low concentrations in both groups. However, among the NC, there was a clear statistical significance of Th1 and Th2 cytokine profiles, with a higher concentration of Th2.

## Discussion

Since the 1990s, a new group of HIV-1 infected persons was detected and they raised new possibilities regarding the outcome of an infection, which was thought to be always fatal [25]. Viremia controllers were initially seen as the natural result of the evolution of host/agent interaction, although a few decades have elapsed since the recognition of the virus and its severe consequences to the human host [23]. Virus evolution and host genetic selection would fit the idea that a new human specific group could control the virus replication without disease development. Understanding the host behavior, the immunological response, genetic characteristics and virus pathogenesis, would certainly help understand how to improve the outcome of newly infected persons and possibly vaccine production [26, 27].

The present information is the first approach to characterize viral controllers in the North of the Amazon region and similar publications in the country are sparse. Demographic informations from the persons investigated were not relevant for their characterization as they were not different from the persons involved in the general epidemic of HIV-1 and AIDS elsewhere in the country. Similar results have been observed in other geographical settings regarding the demographics of viral controllers without statistical significance [28]. The importance of the similarity of the data is of relevance as the population group investigated, is highly miscigenetic [29], contrary to other groups.

The number of HIV-1 viral replication controllers is extremely low anywhere and their detection is a difficult task during the routine clinical approach. The use of statistical methods commonly used in epidemiology when dealing with rare events, was a strong statistical support, which corrected and balanced the low numbers of persons by using all the available measurements of TCD4^+^, TCD8^+^ and VL, along the observation period, and improved the statistical analysis of the numbers presented. VC1 did not show any detectable level of viral multiplication, but VC2 showed blips measured by the plasma viral load (in agreement with their definition in Table 1), which were controlled without the use of ART. It was remarkable to see that the group was able to recover from viral loads up to 10,000 copies, but this is not quite different from definitions [30] which include a limit of 2000 copies (log_10_ = 3,3010). The peak of higher viral loads was more frequently seen in NC as expected. The persistence of the virus in the lymphatics acts as a constant repository for the virus from these reservoir tissues [31]. It is quite certain that the virus is in continuous replication, even among VC1 [16, 17, 32, 33] and it would certainly cause a decline in LTCD4^+^, which was not seen in our VC groups. Cure agendas can not ignore that the groups are different and should be addressed differently in the present suggestions for “test and treat”. The main point of interest is of how long should one go on waiting to consider a person as a viral controller? This is a controversial point [14] and should be addressed with priority.

Levels of LTCD4^+^ were consistently high, along the observation period among the viral controllers and the evolution of the counts showed no decrease, contrary to what has been described [17]. Absolute and relative counts of LTCD4^+^, were commonly used as predictors of disease progression and the recovery of cell counts after ART, a highly relevant variable to monitor the clinical outcome of HIV-1 infected persons [34–37]. The overall annual change in their values along the observation period was not significant, although it was not possible to measure whether the lymphocytes were fully functional or abnormally activated. They should still be useful markers even in the present protocol of ART.

LTCD8^+^ counts were also kept higher and did not change their levels during the observation period in the VC and NC groups. They were constantly stimulated which led to values quite higher than the uninfected controls and did not change along the years. It is still not completely understood the interaction of LTCD8^+^ in the pathogenesis of HIV-1, thus it is fair to think in the increase of the immunological response based in LTCD8^+^, both to protect and to change the outcome of the disease [38–40]. A recent report showed that HIV suppressive activity mediated by CD8^+^ T lymphocytes in vitro was significantly greater among VC than NC cells, with a significant correlation between the suppressive activity and the plasma viral load [41]. It is a regular activity of LTCD8^+^ to keep adequate levels of IFN-γ, an important cytokine in the arrest of viral multiplication, both during the innate immunological response (by natural killer T cells) and by LTCD8^+^ cytotoxic cells [8, 15, 42, 43].

“Non-AIDS related events”, involving chronic disorders of the CNS, the cardiovascular system and cancers are frequently described [44, 45]. Atherosclerosis and coronary heart disease, are commonly observed in an earlier age among HIV-1 carriers and no relevant clinical signs are readily detected by the patient or the attending physician. The inversion of LTCD4^+^/LTCD8^+^ ratio values, is in direct association with the progress towards AIDS and non-AIDS related events and when the inversion is sharp (below 0.5), this simple measurable variable becomes a good biomarker of mortality by other causes than AIDS [46–48] and an apparently reliable marker to trace early heart disease among infected persons virally suppressed under ART [49]. Our results showed that the groups have a clear inversion of the ratio, although in a more subtle level among the viral controllers (VC1 and VC2). The marker did not change with time and the general health of the persons was apparently good. If there is a possibility of using the ratio as a biomarker, it will certainly require the definition of a cut off to establish an appropriate moment to start looking for other chronic disorders among non-controllers and the adequate moment to intervene with viral controllers as well. Apparently, there is no other published similar result considering the long term decline of LTCD4^+^/LTCD8^+^ ratio among viral controllers, but for the period of observation in the present study, the decrease in the inversion of the ratio was not enough to be significant in the VC group.

The presence of CCR5∆32 and SDF1–3’A polymorphisms could not be associated to a better response against virus multiplication and control by the host, but the number of persons involved in the present study clearly did not allow an appropriate statistical evaluation. Our previous data showed that these polymorphisms are not usual among HIV-1 infected persons in the North region of the country [50]. Although some polymorphisms are strongly associated with viral controllers, HLA subtypes were also not associated with differences in the cytokines and chemokines production between elite controllers and viremic controllers regarding the frequencies of the alleles B27 or B57 [17, 33], contrary to what is generally accepted [26, 51–54]. In the present study, the evolution in the counts of LTCD4^+^ and LTCD8^+^ were not influenced by both polymorphisms. Larger studies will be necessary to confirm or not the influence of the allele 3’A in the low counts of LTCD4^+^ and LTCD8^+^. Despite the absence of statistical significance there is an indication for the possible involvement of M-tropic, using CCR5 related co-receptors and T-tropic isolates, using CXCR4 [55, 56]. Most of these studies with polymorphisms were performed using samples from non-viral controllers with a regular progression to AIDS; viral controllers are still poorly understood regarding these and other polymorphisms.

IFN-γ was the sole cytokine presenting a higher level in the VC group. The relevance of IFN-γ resides in its ability to inhibit viral replication. The maintenance of regular levels of this cytokine is a possible consequence of LTCD8^+^ activity. These cells have an important role in the expression of IFN-γ both in the innate immunological response (by natural killer T cells) and by CD8^+^ cytotoxic T lymphocytes once antigen-specific immunity develops [8, 42]. IFN-γ is also an inducer of inflammatory chemokines (MIG and IP-10) which are responsible for the recruitment of leucocytes to the inflammation site and act in the chronic immune activation [56–58]. Comparisons have been drawn among different groups. The chemokines were described in lower levels among elite controllers (named VC1 in the present study) in comparison to other groups capable of viral replication control without ART (named as VC2 group), HIV-1 negative volunteers and HIV-1 persons under ART, but it was coherently raised among those regular progressors [10, 12, 17]. It was not possible to show in our work 7or in any other, the extension of the inflammatory response, and considering the low levels, IFN-γ is probably more involved with control of viral replication, as we are dealing with a persistent and active viral infection. Most of the time it is shown that LTCD8^+^ producing IFN-γ are weak responders among the VC and their levels of production gradually increases among regular progressors under ART [31, 53, 59]. Inflammation markers are a common finding [60, 61] in the cases of treatment interruptions and the objectives of ART with the new protocol intend to suppress virus multiplication, prevent inflammation and provide an effective immune response.

Th1 and Th2 cytokine profile between the VC and NC groups, made it possible to observe the higher plasma concentration of Th2 cytokines in the NC group and Th1 cytokines in the VC. The immune response centered in Th1 cytokines reflects a non-progressive HIV-1 disease, but it may indicate a probable helper protection against HIV-1 infection promoting phagocytosis, stimulating NK cells, T lymphocytes and a consequent elimination of the virus, suggesting a low grade inflammation, persistence and low level viral replication. The exacerbation of Th2 cytokine profile leading to an increased humoral response, is not sufficient for the containment of intracellular infectious agents, contributing to viral multiplication and a faster progression to AIDS [62–64]. The down regulatory effect of IL-10 on Th1 cytokines was not evident among VC; indeed, the shift observed here from Th1 to Th2 cytokines is related to progression of HIV-1 disease. IgG antibody response to HIV-1 gradually increases from viral controllers to non-viral controllers [33, 39, 65–67] which is in clear agreement with this Th1 to Th2 signature change presented here. Our results point to a distinct hypothesis proposed [17, 68] who associate the AIDS progression to the continuous inflammatory environment. It is possible to suggest that an equilibrium of the cytokine Th1 synthesis, especially the IFN-y, could be more favorable to the VC regarding the activation of the cellular immune response, an important step against to impair viral replication.

Numerous comparisons have been drawn among the different groups, including elite controllers, viremia controllers, HIV-1 progressors (successfully being treated or not) and HIV-1 negative volunteers [10, 12, 17], but the definition of viral controllers was not consistent in the past [25, 69, 70] and it continues in the present. The description of this new and rare phenotype of HIV-1 infected persons, so far, has not elicited a successful characterization of the group and a large number of proposed definitions continue to impair a better understanding not only of the real prevalence numbers of the controllers, but also, of the biological mechanisms involved in the control of the virus, including those so called elite controllers [7–10, 12, 14, 71–75].

The recent suggestion for a new ART protocol [19–21], is also making us face a continuous and worrying increase in the adoption of the protocol to treat as soon as one discover his/her status of HIV-1 infection [37]. This will also soon hide all the chances to understand how the infection is modulated by the host in such a rare and special situation. Adherence to rigid criteria in the definition of viral controllers is still not attainable. The variables considered and the values taken into consideration with each of the groups, is far from a consensus. Laboratory variables, including immunological and virological ones, time of known infection, time of maintenance of virus under replicative control and decrease of host cell destruction in the absence of ART, should be more strictly followed. It is not possible to continuously face self-adequate, home made, laboratory, institution or country definitions for viral controller or elite controllers. It is relevant to mention that the conclusion of a large cohort study indicates that interruption of ART started at the early stages of infection is apparently not detrimental to treatment in the chronic phase of the disease [76].

## Conclusions

The knowledge of viral controller characteristics in different population groups, including genetic background, is important to define an strict universal definition for the sake of learning about the pathogenesis of HIV-1. It is required to adhere to long periods of observation without ART, regular counts of LTCD4^+^ and LTCD8^+^ and undetected viremia. This will be the only possible way to compare such important information in order to understand the mechanisms which lead to the control of viral replication. Data on LTCD4^+^ seems to be stable and repetitive from published data, but the LTCD8^+^ response and the significance of LTCD4^+^/LTCD8^+^ ratio values are in need to further exploration as biomarkers. The change from Th1 to Th2 cytokine profile may help to design and adjust specific treatment protocols for the group.

## Abbreviations

ART: Antiretroviral therapy
CAGR: Compound annual growth rate
CCR5: C-C chemokine receptor type 5
CNS: Central nervous system
IFN-y: Interferon-gamma
IL: Interleukin
IP-10: IFN-gamma-inducible protein 10
LTCD4^+^: T CD4^+^ lymphocyte
LTCD8^+^: T CD8^+^ lymphocyte
MIG: Monokine induced by interferon-gamma
NVC: Non-viremia controllers
SDF1: Stromal cell-derived factor-1
Th: T helper cells
VC: Viremia controllers
